# The Effectiveness of Nudging and Its Ethical Implications

**DOI:** 10.1111/bioe.70000

**Published:** 2025-06-15

**Authors:** Leonard Dung

**Affiliations:** ^1^ Institute for Philosophy II Ruhr‐University Bochum Bochum Germany; ^2^ Centre for Philosophy and AI Research University Erlangen‐Nürnberg Erlangen Germany

**Keywords:** autonomy, manipulation, nudging, publication bias, rationality

## Abstract

Nudging consists of interventions that aim to alter behavior in a certain way by changing the presentation or framing of options, without coercion or changing economic incentives. This paper discusses the effectiveness of nudging and the ethical implications of this effectiveness. Section 2 suggests that—if publication bias is adequately accounted for—recent comprehensive meta‐analyses as well as high‐quality experiments show that nudging is much less effective than previously assumed. Sections 3 and 4 discuss the ethical implications. I argue that the lack of effectiveness of nudging is an additional moral consideration against it. There are two reasons: First, reduced effectiveness makes nudging less cost‐effective. Second, reduced effectiveness reduces the benefits of nudging but does not, to the same degree, weaken the moral reasons speaking against nudging. However, a comprehensive assessment of the effectiveness of various forms of nudging in diverse contexts, as well as their ethical permissibility, requires further empirical and ethical research.

## 1 Introduction

According to the canonical definition of Thaler and Sunstein [1], a nudge is “any aspect of the choice architecture that alters people's behavior in a predictable way without forbidding any options or significantly changing their economic incentives.” The choice architecture consists of how options are presented or framed. So, nudging interventions aim to change behavior by changing the presentation or framing of options, without coercing or changing economic incentives.^1^ Examples of nudges are as follows:

1. Defaults. Determining that, upon their death, people have consented to donating their organs by default, i.e., unless they explicitly opted out [2, 3].
2. Reminders. Sending people a reminder to schedule a check‐up with the dentist [4].
3. Physical positioning. Presenting healthy food in the cafeteria at eye‐level to increase its saliency [5].

As these cases illustrate, nudging can occur in a wide variety of domains. In this paper, I will talk about nudging generally, not in a specific domain (e.g., medical practice), since most of the relevant arguments generalize between contexts. Nudging is different from merely informing or persuading [6] on the one hand, and coercing on the other. For example, *Physical Positioning* does not give people information about the healthiness of food, but it also does not force them to eat healthy food. Nevertheless, it is supposed to make them more likely to order healthy food [5].

Nudging has been praised because it promises to cheaply influence citizens' behavior in substantially beneficial ways, for example, making them eat more healthily or donate more organs, while respecting their freedom. Consequently, it has been taken up by policy‐makers [7, 8].^2^ At the same time, ethicists have discussed nudging controversially. The most influential concern of critics is that nudging might undermine human autonomy or amount to manipulation [10, 11, 12, 13, 14], but we will discuss others in Section 4.

In this paper, I will discuss an objection to nudging that is distinct from its putative violation of autonomy and other concerns in the literature. This objection is simply that nudging may be much less effective than previous ethical evaluations assumed. In Section 2, I will argue that recent evidence suggests that the efficacy of nudging may have been significantly overestimated. In Section 3, I will explain why the ethical permissibility of nudging is related to its effectiveness. Section 4 will show why, in light of recent debates on the ethics of nudging, it is far from obvious that reduced effectiveness provides a moral consideration against nudging. Nevertheless, I will conclude that it is, on balance, plausible that the lack of effectiveness of nudging provides an additional consideration against the ethical permissibility of nudging. I will also elucidate which factors this assessment depends on.

## 2 Nudging and Publication Bias

Adherents of nudging have built up a large pool of studies that have been taken to support the claim that nudges can have strong effects on behavior [1, 15]. However, since the early 2010s, the so‐called “replication crisis” has shaken the social sciences, with behavioral science becoming one of the central targets [16]. In this “crisis,” it was discovered that a surprisingly large number of experiments in the social sciences do not give the same results when they are repeated, that is, they do not replicate [17, 18]. Thus, the effects they claimed to show often do not exist or are much smaller than assumed. In this context, it is natural to ask whether nudging might have smaller effects than assumed, or even no effect at all.

However, a claim about nudging's effectiveness, of course, needs to be grounded in evidence about nudging specifically, not only the replication crisis generally. I will argue that there is indeed credible evidence that nudging might be much less effective than thought. This evidence rests on two sources, which I will detail below. Most of these studies have also been summarized in a blog post by Ritchie [19], which I encourage readers to consult for an accessible overview.

The first source is meta‐analyses. The most recent and comprehensive meta‐analysis of the effectiveness of nudging has been performed by Mertens et al. [20], who base their analysis on over 200 studies that report over 440 effect sizes. At first sight, their result seems to conflict with my claim here. They report an average effect of nudging interventions of Cohen's *d* = 0.43. While this is often considered a small to medium effect, “[i]n the context of behavioral interventions, or even many medical treatments, though, it is substantial: an equivalent would be an intervention that boosted someone's IQ (SD [standard deviation] = 15 points) by six and a half points, for instance (or their height—SD ≈ 6 cm—by about 2.5 cm)” [19]. However, this number should not be taken at face value.

There is evidence that the academic literature on nudging, which the meta‐analysis by Mertens et al. relies on, is pervaded by publication bias. In other words, studies on nudging are more likely to be published when they find statistically significant positive effects, perhaps particularly so if these effects are large. If so, the experiments whose results are published are a biased sample of all the experiments conducted, which leads to overestimates of the effectiveness of nudging. For this reason, Mertens et al. statistically correct for a moderate publication bias, which attenuates the average effect size to *d* = 0.31. However, several commentators argue that their data provides evidence of *severe* publication bias, and that the overall effect vanishes completely or almost completely once this bias is adequately corrected for [21, 22, 23]. For instance, Maier et al. [21] conclude that “when this publication bias is appropriately corrected for, no evidence for the effectiveness of nudges remains.” In response, Mertens et al. [24] wrote a remarkably conciliatory reply that does not dispute any of the substantive objections of the commentators.

Another slightly smaller (100 primary publications) and still quite recent meta‐analysis also notes publication bias as a grave problem [25]. The authors do not run any statistical corrections for publication bias, but “conclude that the findings of this study have to be interpreted with great care and are [sic!] rather represent an upper bound of the effectiveness of nudging” [25], (p. 54). So, at the very least, this analysis is unable to support optimistic claims about the effectiveness of nudging. So, the most recent and most comprehensive meta‐analyses suggest overall that nudging is of limited effectiveness.

Second, DellaVigna and Linos [26] compare the effectiveness reported in experiments of nudging from the published academic literature with 126 randomized controlled trials by two of the largest Nudge Units in the United States. Crucially, the authors have information about all experiments performed by these Nudge Units, so that there is no publication or selection bias in evaluating the results. Moreover, the experiments by the Nudge Units have a much higher average sample size (10.006 vs. 484 participants) and thus much more statistical power (minimum detectable effect size of 0.8 vs. 6.3 percentage points). They find that, in the academic literature, the average reported impact of a nudge is very large (8.7% expressed as the percentage difference between treatment and control groups). By contrast, in the Nudge Unit trials, the average effect is much smaller (1.4%), albeit statistically significant. The researchers argue that publication bias and the higher statistical power of the Nudge Unit trials account for the difference between the average effect in both kinds of trials.

Combined, I take this to be strong evidence that nudging has a much smaller average effect than earlier discussions assumed. Nevertheless, we should not conclude that nudges generally have no effect. First, this stronger claim would require more evidence. Second, DellaVigna and Linos actually find a statistically significant, albeit small, average effect. Third, and most importantly, the term ‘nudging’ encompasses a broad and heterogeneous class of interventions. The meta‐analyses we considered find a huge variability in the effects of different kinds of interventions, and this variability is also what we would expect a priori. On the face of it, there is not much reason to expect that different nudging interventions, ranging from defaults to encourage organ donation to small changes in presentation of food to cause healthier eating, would have similar effect sizes (see also [19]). Thus, some nudges might have big effects, even if most have small or no effects.

However, importantly, there is currently significant uncertainty about which nudges tend to be highly effective [22, 27]. The best way to make estimates about the effectiveness of nudges in different domains is probably to use methods to statistically correct for publication bias and to rely on experiments which are not subject to publication bias [26], (sect. 4.4). Yet, with the first method, some caution is warranted, since false assumptions about the extent of publication bias in a domain would lead to misestimates. Moreover, the trustworthiness of meta‐analyses of nudging studies is also constrained by factors besides publication bias. In particular, studies on nudging are highly heterogenous on many dimensions, for instance target populations, experimental design and outcome measures, making averaging the results of different studies nontrivial [27] (p. 362).

Since the point I will be interested in has to do with the ethics of nudging generally, I will simplify by speaking of the effectiveness of nudging interventions *in general*. This is compatible with the view that we may have evidence that some particular nudging interventions are highly effective. In general, a priority for future research should be to measure more robustly which specific types of nudges tend to be very effective, and which have less of an impact.

If the preceding studies, which constitute the best current empirical evidence on the effectiveness of nudging, paint a roughly correct picture, then previous discussions of nudging, including the ethics of nudging, have often relied on over‐inflated estimates of their effectiveness. From now on, I will assume that nudging is indeed much less effective than previously assumed and discuss the ethical implications of this observation. In accordance with most of the ethical literature, I will focus on nudges, which aim to cause behavior that is in the nudged subject's interest, and mostly on nudges by the government.^3^

## 3 The Ethical Permissibility of Nudging in the Context of Its Effectiveness

I will investigate whether the (lack of) effectiveness of nudging provides an additional consideration against the ethical permissibility of nudging. In other words, if nudging is less effective than we thought, should this leave us more, less, or equally skeptical of the ethical permissibility of nudging? This question is relevant, no matter how one antecedently views the ethical status of nudging. So, I mostly remain neutral on controversies regarding the ethics of nudging from the literature. I only claim that, on a wide range of views of the ethics of nudging overall, our assessment of the effectiveness of nudging provides an *additional* consideration against the ethical permissibility of nudging. Which assessment of the *overall* ethical status of nudging is correct will depend on the evaluation of all the different ethical arguments discussed in the literature.

The first reason for my claim is directly grounded in the cost‐effectiveness of nudging, while the latter discussion raises more controversial philosophical issues. First, nudging is advocated for because of its presumed cost‐effectiveness. The promise is that nudging provides more health benefits, donor organs, etc. (whatever the aim of the intervention is) per dollar spent than alternatives. If nudging is less effective, it is less cost‐effective. Thus, some interventions that seemed cost‐effective will turn out not to be cost‐effective once we consider recent evidence suggesting that nudging might be less effective than thought. If governments are not allowed to waste taxpayer money, then interventions which are not cost‐effective (compared to alternative interventions which are not worse in other respects) are impermissible.^4^ In sum, evidence of lower effectiveness of nudging speaks against its cost‐effectiveness, which, all other things being equal, provides an additional consideration against the moral permissibility of nudging.^5^

Note that this view does not presuppose ethical consequentialism, that is, the view that the moral permissibility (and obligatoriness) of actions depends *only* on their consequences. It only depends on the much weaker view that the moral permissibility of actions depends *partly*, potentially among many other considerations, on their consequences.

It is also important to consider that a nudge that is effective in producing the intended behavioral change may not always lead to welfare benefits overall. For example, a nudge that successfully encourages healthier eating may also impose additional welfare costs by causing people to eat food they like less [28].^6^ The latter might reduce people's welfare. In principle, it is an open question when the health (or other) benefits caused by nudging exceed additional costs that nudging might impose. Moreover, there may not be much variation between people in how much their welfare benefits from a nudging intervention [9] (pp. 96–99).

This opens up the possibility that, in some cases, reductions in the average effectiveness of nudging stem mostly from reduced effectiveness in nudging people whose welfare would not increase (all things considered) due to the nudge (e.g., because these people have strong preferences against the option they are being nudged toward). I will set aside these cases here and focus on the ethical evaluation of cases where nudging's decreased effectiveness reduces welfare benefits.

This is because, in cases where reduced effectiveness of nudging leads to higher welfare, the ethical assessment seems quite simple: It is plausible that, in these cases, reduced effectiveness would provide an additional consideration in favor of, not against, the moral permissibility of the nudging. The reason is that in these cases, reduced effectiveness leads, by assumption, to higher overall welfare and has no offsetting disadvantages. So, when I talk about reduced effectiveness of nudging in what follows, I only intend to refer to reductions that proportionally decrease the welfare benefits of nudges.

Of course, my claim that reduced effectiveness of nudges provides an additional consideration against the moral permissibility of nudging does not imply (by itself) anything about when nudging is morally impermissible (or permissible). Even if nudging is less effective than thought, one may still maintain that many nudging interventions are cost‐effective. Part of the reason is that nudges are often very cheap (e.g., changing the position of food items in the cafeteria), that is, have low costs. One may also reply that some nudges may be very effective, even though the average effectiveness of nudges is smaller than thought. This is true. Yet, the significance of this reply is limited. If nudges are much less effective on average, then many specific nudges must be much less effective.

However, most of the ethical discussion of nudging does not center on its cost‐effectiveness. Instead, the central question is whether nudging might be morally impermissible, even if it is cost‐effective (i.e., it evaluates nudging without considering opportunity cost). This might be, for instance, if nudging involves problematic manipulation or undermines human autonomy. If such concerns entail a categorical prohibition of (some forms of) nudging, then the cost‐effectiveness of (these forms of) nudging is beside the point. If these concerns speak against nudging but can be outweighed if the benefits of nudging are sufficiently big, then cost‐effectiveness considerations are relevant.

I take it to be plausible that few, if any, of the forms of nudging which are controversially discussed in ethics are categorically prohibited. This is because it is plausible that even actions which break strong moral rules (e.g., against stealing very valuable items) can be permissible if the moral stakes are sufficiently high (e.g., millions of human lives).

For most forms of nudging, which are controversially discussed in ethics (e.g., defaults), it is obvious that they violate weaker moral rules than stealing. However, this may not apply to all cases, for example, when a physician nudges their patient to perform a life‐changing operation. One may argue that, in such a case, nudging is categorically prohibited. If so, then, in these cases, cost‐effectiveness considerations are irrelevant.

If all this is correct, then, in most ethically interesting cases, nudging is not categorically prohibited.^7^ If so, whether nudging is permissible in any given case plausibly depends on whether the use of nudging is proportionate, given its expected benefits. Suppose nudging compromises important ethical values or principles. I will call moral concerns of this general kind the ‘moral harm’ of nudging. If the benefits of nudging are too small or can be achieved in less ethically problematic ways, then nudging is impermissible. However, if the benefits of nudging are sufficiently high and cannot be achieved in alternative ways, then nudging may be a proportionate means to achieve these benefits and thus be morally permissible.

## 4 Scrutinizing the Decreased Harm Objection

If so, then the following conditional holds: If nudging is less effective than often assumed, then this provides an additional consideration against the moral permissibility of nudging. So, we would need to critically reevaluate in which contexts the use of nudging is morally permissible. I think that this condition is true. However, there is an important objection, which I discuss now. Arguably, if nudging is less effective, then it is also less morally harmful. For saying that nudging is less effective means saying that it is a less strong influence on peoples' behavior. Arguably, this means that nudging is less manipulating, or a smaller threat to human autonomy than otherwise. Call this the *decreased harm objection*.

For an argument in line with this objection, consider that some proponents of nudging suggest that nudges should be “easily resistible” [29] (p. 489) or “easy and cheap” to avoid [1] (p. 6). This entails that “Arguments for nudging require it to be effective, but make it too effective and you might end up worrying about resistibility” [12]. According to this thought, nudging aims to pick a sweet spot of effectiveness; it may be *too* effective. This opens up the possibility that the evidence we have considered may even be good news for the ethical permissibility of nudging. For all we know, perhaps a decrease in the effectiveness of nudging brings it closer to this sweet spot and thus makes it more likely to be morally permissible.

However, I argue now that the decreased harm objection is likely false. The objection depends on the assumption that reductions in the effectiveness of nudging reduce its moral harm in proportion to its benefits, so that nudging is equally likely to be a proportionate course of action.^8^ However, most accounts of the moral harm of nudging in the literature entail that this assumption is not fulfilled. Based on the overview of the ethics of nudging by Schmidt and Engelen [12], I will mention six reasons, commonly discussed in the literature, why nudging may be morally problematic.

1. Nudging is untransparent, and thus removed from adequate individual and democratic control, such that it constitutes the exercising of illegitimate control of governments over their citizens [30, 31].
2. The emphasis of nudging on individual choice might distract from deeper structural causes of societal ills, and thus impede changes to them [32, 33].
3. Nudging is (at least in the context of a doctor obtaining consent from a patient for a medical intervention) incompatible with the requirements for genuine informed consent, since such informed consent requires that the doctor tells “the truth, the whole truth, and nothing but the truth” [34] about the benefits and risks of the medical procedure [35]. On this view, nudging is not truth‐telling in the relevant sense. Instead, Simkulet argues, it is often better understood as bullshitting in the sense of Frankfurt [36].
4. Nudging (when done by the government) involves the government's imposition of values on its citizens and may thus be impermissible [37].
5. Nudging might fail to respect or even undermine the rationality of humans subjected to it, because it influences decisions via irrational (or arational) processes [10, 38, 39].
6. Nudges influence our choices such that the resulting actions no longer reflect our genuine desires, compromising our volitional autonomy [12]. This is related to the view that nudging may undermine our agency, since it infringes upon active choice which is necessary to freely form one's own preferences and, thus, oneself [40].^9^

Assume these views are correct. The question at issue is then conditional: If nudging turns out to be less effective and one of these views is true, do the moral reasons against nudging, that is, the putative moral harm of nudging the view specifies, decrease in strength, and by how much? Let us go in order:

1. Moral reasons against nudging purely based on transparency concerns are independent of the effectiveness of nudging: Such reasons do not become (significantly) weaker because evidence of nudging's effectiveness is (normally) irrelevant to how transparent it is.

2. The extent to which the emphasis on nudging distracts from structural problems of society does not depend on the effectiveness of nudging, but it may depend on beliefs of policymakers and the public about the effectiveness of nudging. For the purposes of my argument, it is sufficient to observe that new evidence that nudging is less effective than thought does not by itself give us evidence that talk about nudging is less of a distraction from structural problems than we thought. So, by itself, the reduced effectiveness of nudging does not weaken this moral reason against nudging. However, second‐order effects, in which new evidence on the effectiveness of nudging changes the public perception of nudging, are important and should be considered.

3. According to 3, nudging violates obligations for truth‐telling, which speaks against the moral permissibility of nudging. It is plausible that the strength of the moral reason for truth‐telling does not change much, even if the effectiveness of the nudge changes. On this view, that nudging violates moral obligations for truth‐telling would still significantly speak against it, even if the nudging intervention were completely ineffective. This shows, at a minimum, that the strength of this moral reason against nudging does not decrease proportionally to its effectiveness.

4, 5, and 6. These three moral reasons against nudging can be understood in two ways. On a *subjective* reading, the relevant moral harm of nudging is based on the presence of an intention or an attempt to change behavior via nudging. On this reading, for example, there is something morally wrong with the government *trying to* impose its values on citizens, independently of its change of success, and there is something disrespectful about merely trying to influence decisions via irrational processes. On an *objective* reading, the relevant moral harm of nudging requires that the nudging intervention successfully changes behavior. It may require that the government successfully imposes its values, that decisions are actually influenced irrationally, and that agency is actually undermined. An objective reading supports the decreased harm objection, while a subjective reading does not. I think that objective and subjective readings of these objections are both somewhat plausible and may be favored by different proponents of these objections.

I hold that, in most researchers' views, the moral harm of nudging is partly based on its subjective and partly on its objective component. To test the plausibility of this view, we can imagine cases where a nudging intervention has not only zero costs (monetary or other welfare costs) but also no effect on behavior. So, imagine someone uses a nudging intervention which constitutes an attempted imposition of the government's values or an attempt at irrational influencing human decision‐making or undermining human agency. However, the nudge predictably has no effect.

It seems plausible to me that the relevant moral harm (value imposition, irrational influence on values, or undermining of human agency) is significantly weaker in this case than when the nudge has a chance to actually change behavior. On the other hand, it still seems plausible to me that, if these objections against nudging are sound, the reasons mentioned against nudging—or at least reasons 4 and 5—still provide some moral reason to not engage in nudging, even if it predictably does not change behavior. It is plausible that performing the nudging intervention would still be wrong and cause some degree of moral harm, on these views.

If the moral reasons against nudging, on these views, are partly based on its subjective component, then, at least, the moral reasons against nudging do not lose weight fully in proportion to its effectiveness. This is independently plausible, since the view that the moral reasons against nudging lose strength fully in proportion to its effectiveness would be committed to the following claim: If a nudging intervention I1 is ten times as effective as another one I2, then—all other things being equal—the relevant moral reason against I1 (imposition of values etc.) is ten times as strong.^10^ Yet, it may seem implausible that the difference in the moral harm of both interventions is as strong. Yet, if moral reasons against nudging do not lose strength fully in proportion to their effectiveness, then this seems to suggest that, on balance, reduced effectiveness of nudging decreases its benefits more than it decreases its (potential) moral harms. However, I grant that these considerations are not conclusive, and that a view on which the moral harm of nudging specified by 4, 5, and 6 does decrease in proportion to its effectiveness can also reasonably be defended. Moreover, other views on the moral harms of nudging—which have not been discussed here—may come to a different verdict on the question whether the moral harms of nudging depend on their effectiveness.

To summarize this section: We have discussed the decreased harm objection, which depends on whether different putative moral reasons against nudging become weaker if, and to the extent that, nudging is less effective. For reasons 1 and 2, it is plausible that they do not become significantly weaker if nudging is less effective. For 3, the reason may become less strong, but it retains significant strength even if nudging had no effect. For 4, 5, and 6, the matter is much more uncertain. However, I have argued that, even though these reasons against nudging significantly lose in strength with decreasing effectiveness, their loss in strength is likely not proportional to reductions in effectiveness. If so, reduced estimates of the effectiveness of nudging decrease its expected benefits more than they decrease its moral harms. If so, evidence of low effectiveness of nudging provides an additional moral consideration against it, despite the decreased harm objection.

In conclusion, Section 2 has shown that there is credible evidence that nudging is much less effective than previous discussions of the ethics of nudging assumed. Sections 3 and 4 have argued that this provides an additional moral consideration against nudging. There are two reasons: First, reduced effectiveness makes nudging less cost‐effective. Second, reduced effectiveness reduces the benefits of nudging but does not, to the same degree, weaken the moral reasons speaking against nudging. However, a comprehensive assessment of the effectiveness of various forms of nudging in diverse contexts and their ethical permissibility requires further empirical and ethical research.

## Data Availability

The study collects or uses no data of its own.

## Endnotes

1 I say that nudging interventions “aim to change” behavior, rather than they in fact alter it, because I do not want to preclude by definition that there could be ineffective nudging interventions.

2 For the case in favor of nudging, see Thaler and Sunstein [1] and Sunstein [9].

3 Many potential cases of nudges which are not performed by the government (e.g., by physicians to patients) allow for a corresponding government case where similar considerations apply: for example, a government program which promotes this nudging behavior or a government‐led organization which teaches to perform this behavior.

4 One may object that some nudges have no monetary cost at all, for example, physicians nudging patients to accept interventions which are (allegedly) in their interest. In such cases, we could just talk about “effectiveness” and ask whether reduced effectiveness of nudging would speak against its moral permissibility. However, I think that such cases will be very rare. For example, this intervention may still have some nonmonetary cost, for example, effort for the doctor. Moreover, cost‐effectiveness considerations are entered at least on the policy level, for example, when thinking about whether to train new doctors to nudge their patients.

5 As discussed later in this section, views according to which nudging is categorically prohibited entail that cost‐effectiveness considerations are irrelevant to the ethical permissibility of nudging.

6 Other examples are possible: Most importantly, the nudging intervention might cause side effects—not directly related to the behavior being nudged—to the person being nudged or to third parties.

7 That being said, nudging might be prohibited in almost all situations that agents realistically face, which would make considerations of the effectiveness of nudging mostly irrelevant.

8 Another theoretical option is that reductions in the effectiveness of nudging reduce its moral harms even more than its benefits, but it is not clear why this would be true.

9 For reasons of space, this is merely a rough gloss of Schubert's nuanced theory.

10 I am assuming here, for simplicity, that effectiveness corresponds to welfare benefits (see above).
